# Antifungal Activity of *Eclipta alba* Metabolites against Sorghum Pathogens

**DOI:** 10.3390/plants8030072

**Published:** 2019-03-22

**Authors:** Rajini Sollepura Boregowda, Nandhini Murali, Arakere C. Udayashankar, Siddapura R. Niranjana, Ole S. Lund, Harischandra S. Prakash

**Affiliations:** 1 Department of Studies in Biotechnology, University of Mysore, Manasagangotri, Mysuru 570006, India; rajinis16@gmail.com (R.S.B.); mnandhini2606@gmail.com (N.M.); ac.uday@gmail.com (A.C.U.); niranjanasr@rediffmail.com (S.R.N.); 2 Department of Plant and Environmental Science, University of Copenhagen, HøjbakkegårdAllé 13, 2630 Taastrup, Denmark; osl@plen.ku.dk

**Keywords:** *Eclipta alba*, eclalbasaponin, sorghum pathogens, MIC, mass spectrometry

## Abstract

Unscientific use of synthetic fungicides in plant disease management has environmental ramifications, such as disease resurgence and serious health problems due to their carcinogenicity. This has prompted the identification and development of eco-friendly greener alternatives. *Eclipta alba* extract was evaluated for its antifungal activity in in vitro and in vivo against sorghum fungal pathogens *Fusarium thapsinum, Alternaria alternata, Epicoccum sorghinum,* and *Curvularia lunata*. The column purified methanolic extract of *E. alba* exhibited good antifungal activity against the target pathogens. The MIC was observed at 80 mg/mL for all tested pathogenic fungi, whereas MFC was 80 mg/mL for *E. sorghinum*, 100 mg/mL for *F. thapsinum*, *A. alternata,* and *C. lunata.* In vitro germination percentage was significantly high in seeds treated with *E. alba* extract (98%) over untreated control (91%). Significant disease protection of 95% was observed in greenhouse and 66% disease protection was noticed in field experiments. The efficacy of *E. alba* extract in field conditions was improved with the use of *E. alba* extract formulation. The profile of phytochemicals in *E. alba* methanol fractions was obtained by ultra-performance liquid chromatography (UPLC) mass spectroscopy. The [M-H]^−^ at *m*/*z* 313.3, *m*/*z* 797.9, and *m*/*z* 269.0 revealed the presence of wedelolactone, eclalbasaponin II, and apigenin, respectively. The H-nuclear magnetic resonance spectroscopy (^1^H-NMR) chemical shift value supported the findings of the mass spectrometry. The results highlighted the possible use of *E. alba* methanolic extract as alternative to chemical fungicide in sorghum disease management.

## 1. Introduction

The unmethodical usage of chemical fungicides for plant disease management has ecological implications, such as disease resurgence due to development of resistance in the target pathogens, and poses pollution problems. The repeated use of synthetic fungicides led to resistance in target pathogens with serious health problems in humans and animals due to their carcinogenicity, teratogenicity, and acute toxicity [1]. This has prompted a need for the investigation of alternative measures for crop disease management. So far, relatively very few botanical formulations have been registered and commercialized. The pharmacological and antimicrobial importance of aqueous and solvent extracts of *Eclipta alba* has been reviewed extensively [2,3]. The safety of *E. alba* extract was confirmed by preclinical toxicology studies using acute oral toxicity, eye irritation tests, and dermal irritation on New Zealand white rabbits and Sprague dawley rats [4]. 

*Eclipta alba* (L.) is an annual herbaceous plant, commonly known by the name ‘False daisy’ placed under Asteraceae family. *E. alba* is extensively used in Indian the traditional ayurvedic medicine system. *E. alba* has been used for the treatment of skin diseases, liver diseases, hair treatment and also as an antimicrobial, analgesic, anti-haemorrhagic, anti-hyperglycemic, and antioxidant agent [5]. *E. alba* contains a wide range of active metabolites, such as coumestans, triterpenoid saponins, flavonoids, alkaloids, and a glucoside of a triterpenic acid [6].

Sorghum is an important staple crop of semi-arid regions of world, especially in India and Africa. Grain mold caused by *Fusarium thapsinum*, *Alternaria alternata, Epicoccum sorghinum,* and *Curvularia lunata* is a destructive disease in sorghum. The pathogenic fungi also spoil the nutritive value of the grains by producing mycotoxins [7]. The fungal infection leads to heavy loss in grain yield as well rendering the grain unfit for human consumption [8,9]. The fungal infection mainly results in seed discoloration, loss in seed germination, seedling growth and overall field stand [10]. Aflatoxin, ochratoxin, and zearalenone are the major mycotoxins contaminating sorghum seeds [11]. Zearalenone, fumonisins, trichothecenes, and fusariotoxins produced by *Fusarium* spp. cause an adverse impact on human health. Zearalenone has been reported to have adverse effects on the reproductive system in animals and causes cancer in human. The mycotoxin produced by the genus of *Alternaria* induces mutagenesis in mammalian cells and causes gastrointestinal haemorrhage in animals [12]. The cytochalasins produced by *Curvularia* and *Epicoccum* species inhibit cytokinesis, protein synthesis, and induce brain oedema and pulmonary haemorrhaging in mice [13]. Currently, infestation of these economically destructive pathogenic fungi in sorghum is managed by chemical fungicides. Even though the long-term use of chemical fungicides induces tolerance in pathogens, carcinogenic health risks, toxicity on non-target organisms, and environmental pollution, farmers are inevitably using the expensive chemical fungicides. 

The current study was intended to explore the antifungal potential of *E. alba* in vitro on economically destructive sorghum grain mold pathogens, such as *F. thapsinum, A. alternata, E. sorghinum,* and *C. lunata*. We have also evaluated the effect of *E. alba* extract treatment on in vitro germination and seedling emergence in vivo, and disease incidence and growth parameters under greenhouse conditions. Further, the phytochemicals in antifungal fractions of *E. alba* extract were characterized by ultra-performance liquid chromatography (UPLC) coupled triple quadrupole mass spectrometry (UPLC-MS/MS) and ^1^H-NMR. 

## 2. Materials and Methods

### 2.1. Chemicals

Hexane, ethyl acetate, methanol, and formic acid solvents were procured from Hi-Media Laboratories, Mumbai, India.

### 2.2. Plant Collection and Identification

Fresh matured *E. alba* plants were collected from paddy fields and humid sites with an annual humidity of 75% and insolation 5.45 kWh/m^2^/day in the vicinity of Mysore (12.2958° N, 76.6394° E), Karnataka. The plant was taxonomically identified by Dr. K. K. Sampath Kumara, Taxonomist.

### 2.3. Extraction of E. alba

The aerial parts of *E. alba* were separated from wild plants, washed thoroughly 5 to 6 times with running tap water and shade dried at room temperature for 15 days, then pulverized to a fine powder. Thirty grams of the powdered sample was placed in soxhlet extractor and sequentially extracted with 250 mL each of hexane, ethyl acetate, methanol, and water (50–85 °C). Extracts were concentrated under low vacuum by using rotary evaporator (Buchi Rotavapor, R-205). The dried extracts were collected and stored as aliquots at 4 °C for further analysis.

### 2.4. Isolation of Fungi

The seed borne fungi were detected by the standard blotter method [14]. Farm-saved sorghum seeds originating from the agro-ecological zones of Karnataka were used for the isolation of fungi. The fungal colonies on incubated sorghum seeds were isolated separately, each fungus was further cultured on potato dextrose agar (PDA) plates and incubated at 27 °C for a period of 7 days. The pathogens were identified based on conidial and morphological characteristics. The authenticity of target pathogens was confirmed by Internal transcribed spacer (ITS) sequences Acc. No. *F. thapsinum* KX155571, *E. sorghinum* KY499205, *C. lunata* KY499208, and *A. alternata* KY674986. 

### 2.5. In Vitro Antifungal Activity of Methanolic Extract and Column Purified Fraction

#### 2.5.1. Agar Well Diffusion Assay

Antifungal activity of different solvent extracts was determined by the agar well diffusion method. One hundred microliters of fungal spore suspension containing *F. thapsinum*, *E. sorghinum*, *C. lunata,* and *A. alternata* at 10^6^ spores/mL concentration was spread on solidified PDA medium. The wells were punched using 5 mm cork borer and loaded with 100 μL DMSO containing 20 mg/mL to 120 mg/mL extracts. Wells loaded with solvent alone served as negative control. Positive control include wells loaded with synthetic fungicide Bavistin (1 mg/mL). All the plates were incubated for 7 days and the diameter of inhibition zone of test microorganism was measured in millimeters. All the experiments were performed in triplicate.

#### 2.5.2. Minimum Inhibitory Concentration and Minimum Fungicidal Concentration 

Broth microdilution method was carried out to evaluate the minimum inhibitory concentration (MIC) and minimum fungicidal concentration (MFC) of *E. alba* methanol extract [15]. Potato dextrose broth (PDB) was added with the required concentration of *E. alba* methanol extracts dissolved in DMSO to obtain the range of concentrations from 20 mg/mL to 120 mg/mL. Ten micorliters of pathogen spore suspension (1 × 10^6^ spores/mL) was inoculated to each flask and incubated for 7 days at 24 ± 2 °C. Control flasks with PDB were inoculated only with pathogen spore suspension and neat DMSO. The least concentration at which no visible growth observed was considered as MIC. Fungal spores from the flasks showing no growth were sub-cultured on new PDA plates to determine the MFC. If the inhibition was reversible, then the extract concentration was considered to have no fungicidal action. MFC is the lowest concentration at which no fungal growth was observed on PDA plates [16]. The experiment was repeated twice with three replications per concentration.

#### 2.5.3. Microtiter Plate Assay

The antifungal activity of purified *E. alba* extract was determined using sterile 96 well plates. The cell densities were determined using tetrazolium dye MTT [3-(4,5-dimethyl-thiazol-2-yl) 2,5-diphenyl tetrazolium bromide] at A570 nm. The 12 wells of each row were filled with 200 µl of PDB (24 g/L, Himedia, India) containing 2 × 10^6^ fungal spores/mL and 10 µl of MTT (2 mg/mL). An aliquot of 40 µl from the stock solution of *E. alba* extract was transferred to the wells to obtain a final concentration of 20 µg/mL to 120 µg/mL. The wells that received 40 µl of DMSO served as negative control and wells with Bavistin (1 µg/mL) were considered as positive control. The fungal spores were allowed to settle for 30 min and the plates were incubated for 48 h. The resulting turbidity was measured at A570 nm after 30 min and 48 h at A570 nm using Tecan infinite M200PRO ELISA plate reader [17]. The antifungal activity was expressed in terms of percent growth inhibition and the extract concentration that exhibits 50% growth inhibition is considered as MIC.

$$\mathrm{Percent}\;\mathrm{growth}\;\mathrm{inhibition}\;=\frac{\mathrm{Corrected}\;A570\;\mathrm{nm}\;\mathrm{of}\;\mathrm{control}\;-\;\mathrm{Corrected}\;A570\;\mathrm{nm}\;\mathrm{of}\;\mathrm{the}\;E.alba\;\mathrm{extract}}{\mathrm{corrected}\;A570\;\mathrm{nm}\;\mathrm{of}\;\mathrm{the}\;\mathrm{control}}\times 100$$

where, corrected A570 nm = A570 nm of the culture measured after 48 h − the A570 nm measured after 30 min.

#### 2.5.4. In Vitro Germination Test

The effect of *E*. *alba* extract on germination of sorghum seeds was tested. Three replicates of 100 seeds were inoculated with target pathogen *F*. *thapsinum*, *E*. *sorghinum, A*. *alternata,* and *C*. *lunata* by soaking seeds for 6 h in a solution containing respective fungal spores at 1 × 10^7^ spores/mL concentration. The inoculated seeds were treated with 120 mg/mL *E. alba* extract. The seeds that received only pathogen inoculation, uninoculated seeds, and Bavistin (1 mg/mL) served as controls.

The between paper method was used for evaluating the germination rate. The seeds were placed between two layers of moist paper towels and kept for incubation. The germination percentage of sorghum seeds was calculated after 10 days of inoculation [18].

### 2.6. Greenhouse Experiment

A greenhouse experiment was conducted to evaluate the effect of *E. alba* extract on seedling emergence, disease incidence, and seedling growth parameters. Sixty sorghum seeds were inoculated separately by soaking each in a solution containing 1 × 10^7^ spores/mL of *F. thapsinum, E. sorghinum, A. alternata,* and *C. lunata*. The inoculated seeds were treated with 120 mg/mL *E. alba* extract. The uninoculated seeds, seeds inoculated with only pathogens, and seeds treated with Bavistin (1 mg/mL) were used as controls.

The 20 treated seeds from each treatment were sown in a plastic pot containing a sterilized mixture of red soil: clay soil: compost (1:1:1) proportion collected randomly from experimental plots of the Department of Science (DOS) in Biotechnology, University of Mysore Karnataka. India. The pots were maintained under greenhouse conditions for 10 days at 25 °C to 29 °C. A completely randomized design (CRD) was used with three replications. The seedling emergence and the disease incidence based on the visual symptoms, such as chlorotic straw-colored elliptical lesions on leaves (*E. sorghinum*), pale lesions with dark brownish red borders on leaves (*A. alternata*), wilt and visual stunted growth (*F. thapsinum*) and reddish brown circular spots on the leaves (*C. lunata*), were evaluated at ten days after sowing. The growth parameters, such as shoot length and root length, shoot and root dry weight, were recorded at 30 days after sowing. 

### 2.7. Field Trials

Field trials were conducted during Kharif season (rainy season—July to October) in experimental plots of the DOS in Biotechnology, University of Mysore, India. Sorghum seeds were soaked in different concentrations of *E. alba* extract in a rotary shaker at 25 ± 2 °C for 6 h. The suspended seeds were dried overnight. Seeds were again soaked in a suspension of the pathogen (10^7^ spores/mL) for 3 h and dried overnight. Seeds soaked in distilled water for the 6 h served as negative control. Seeds dusted with Bavistin (3 g/kg seed) served as positive control. The treated seeds were hand sown in the experimental plot consisting of seven rows five meters long, 60 cm distant, and 20 cm of intra-row spacing with two replications per treatment. The seedling emergence was recorded at 10 days after sowing (DAS), and disease incidence was recorded at 30 DAS, and final counts were made at 60 DAS. At maturity, visual disease observation was taken, and the seed weight was measured as the weight of 1000 kernels from each panicle [19].

### 2.8. Formulation of E. alba Fungicide

*E. alba* methanolic extract formulation was prepared to facilitate ease of application, handling, application, uniformity, sustained release, and penetration by using Xanthan gum (1%), suspending agent (8%), Potassium sorbate (0.5%), alkyl phenyl ethoxylate (3%), defoaming agent (0.5%), *E. alba* methanol extract (15%), and the required amount of water. 

### 2.9. Characterization of E. alba Extract

#### 2.9.1. TLC Profiling of Crude Extract

The chromatographic profiling of crude extract was done by thin layer chromatography (TLC). A combination of Hexane: ethyl acetate: formic acid (8:2:0.5) was used as the mobile phase. Samples of the crude extract and reference standard (eclalbasaponin II and wedelolactone) were spotted on to the chromatographic plate (GF 254 60; Merck 20 mm × 20 mm thick, Darmstadt, Germany) with glass capillary tubes and eluted with the mobile phase. Plates were observed under UV 254 nm and photographed. Further, the spots were developed using a reagent mixture containing p-anisaldehyde (2% *v*/*v*) and sulfuric acid (5% *v*/*v*) in methanol. The retention factor (Rf) values of all the spots were determined and tabulated.

#### 2.9.2. Column Purification of Crude Extract

The crude extract was further purified by passing it through a silica gel column. About 4 g methanolic crude was redissolved in 5 mL of methanol and loaded to columns (3.6 cm × 50 cm) packed with activated silica gel (30 g, 60–200 mesh size) using hexane. The column was then eluted sequentially with 100 mL of hexane, ethyl acetate, and methanol at 3 mL/min of flow rate. Twenty-five milliliters of each fraction were pooled and concentrated to 5 mL using a Buchi Rotavapor. The concentrated fractions were checked for antifungal activity against target pathogens using a disk diffusion assay. Fractions which showed antifungal activity were combined and concentrated. The antifungal fraction was further analyzed by ultra-performance liquid chromatography (UPLC).

#### 2.9.3. UPLC-Diode Array Detector (UPLC–DAD)

The chromatographic profiling of crude methanolic extract and the antifungal fraction was done by reverse phase liquid chromatography (Agilent 1290A series) UPLC equipped with a binary pump, automatic injector and an ultraviolet diode array detector (UV/DAD) module. Separation was accomplished on ACQUITY UPLC HSS T3 column (100 mm × 2.1 mm, 1.6 *μ*m, Waters) using Acetonitrile (A) and water containing 0.1% HCOOH (B). The following gradient elution system was used: 0–15 min, 20–30% A; 15–28 min, 30% A; 28–30 min, 30–40% A; 30–38 min, 40% A; 38–45 min, 40–100% A; 45–50 min, 100–100% A; 50–50.5 min, 10% A; 50.5–60 min, 10% A. An injection volume of 5 μL and a flow rate of 0.3 mL/min were adjusted and the column temperature was set at 30 °C. The spectrum was read at 260 nm UV and the chromatograph profiles of compounds are represented.

#### 2.9.4. UPLC Triple Quadrupole MS/MS

The chromatographic characterization of antifungal column purified sample was performed by Agilent 1290A UPLC consisting of a binary pump, a diode-array detector, an auto sampler, and a column thermostat connected to an Agilent 6590 Q3 mass spectrometry via an electrospray ionization (ESI) interface (Agilent Corp., Santa Clara, CA, USA). The UPLC conditions were the same as explained above.

A full-scan analysis in negative ionization mode was conducted, and the spectra were recorded in the range of *m*/*z* 100 to *m*/*z* 1700. The major parameters used were as follows: drying gas with a flow rate of 8.0 L/min, drying gas temperature 350 °C, nebulizer 30 psig, capillary voltage 3500 V and fragmentor voltage 175 V. The peak abundance of the identified mass was recorded. The identification and characterization of prominent compounds were performed based on the fragmentation pattern. 

#### 2.9.5. H-Nuclear Magnetic Resonance Spectroscopy

The H-nuclear magnetic resonance spectroscopy (^1^H-NMR) characterization of the antifungal column purified sample was carried out using ^1^H-NMR Spectrometer (DDR X—400 *m*/*z* Bruker Deltonics, Germany) using deuterated DMSO as a solvent and the functional groups present in the extract were determined.

### 2.10. Statistical Analysis

The analysis of variance (ANOVA) was performed using OPSTAT software. Mean values among treatments were compared by the least significant difference using critical difference (CD) at a 95 percent level of confidence (*p* < 0.05).

## 3. Results

**Figure 1:**
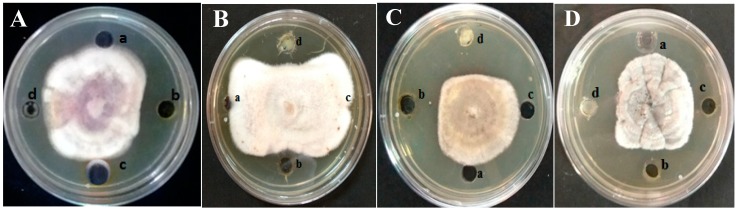
Growth inhibition of (**A**) *Fusarium thapsinum*, (**B**) *Epicoccum sorghinum*, (**C**) *Alternaria alternata* and (**D**) *Curvularia lunata* pathogens by crude methanolic extract of *Eclipta alba* at 120 mg/mL concentration. (a) Control, (b) Methanolic extract of *E. alba*, (c) DMSO, (d) Bavistin.

The present study was designed with the intension of identifying a greener, safer, effective, and economically affordable botanical fungicide. The dried *E. alba* powder was sequentially extracted with hexane, ethyl acetate, methanol, and water using the soxhlet extraction process. The antifungal activity of *E. alba* crude solvent extracts was evaluated by the agar well diffusion method. The methanol extract exhibited the highest antifungal activity with a maximum of 6.0 mm inhibition of *E. sorghinum* at 80 mg/mL followed by *F. thapsinum* (5.8 mm) at 120 mg/mL, whereas *A. alternata* (5.9 mm), and *C. lunata* (5.9 mm) were inhibited at 100 mg/mL. The synthetic fungicide Bavistin at 1 mg/mL showed highest antifungal activity against *E. sorghinum*, followed by *F. thapsinum*, *A. alternata,* and *C. lunata* (Table 1 and Figure 1). 

**Figure 2:**
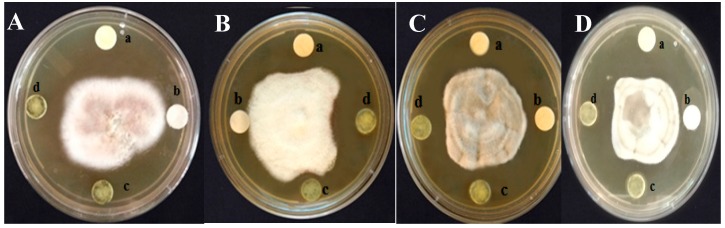
Growth inhibition of (**A**) *F. thapsinum*, (**B**) *E. sorghinum*, (**C**) *A. alternata* and (**D**) *C. lunata* pathogens were observed by column purified fraction of methanolic extract of *E. alba*. (a) Bavistin, (b) Control, (c) Ethyl acetate fraction, (d) Purified fraction of Methanolic extract of *E. alba.*

The crude methanol extract was further purified to differential polarity fractions of hexane, ethyl acetate, and methanol fractions using silica columns and tested for antifungal activity by the agar well diffusion method. The zone of inhibition for *F. thapsinum* was 6.2 mm at 120 mg/mL, 6.8 mm for *E. sorghinum* at 80 mg/mL, 6.6 mm for *A. Alternata,* and 6.5 mm at 100 mg/mL for *C. lunata* (Figure 2). Further, the MIC was carried out by the broth macro-dilution method and the microspectrophotometric method. MIC was observed at 80 mg/mL for all the tested pathogenic fungi. The MFC was 80 mg/mL for *E. sorghinum* and 100 mg/mL for *F. thapsinum, C. lunata,* and *A. alternata.* The MIC determined by the microspectrophotometric method was represented as a percentage of microbial inhibition. 50% growth inhibition for *E. sorghinum, F. thapsinum, C. lunata,* and *A. alternata* was observed at 60 mg/mL of *E. alba* extract (Table 2). 

The germination percentage of the infected seeds upon treatment with *E. alba* extract was 98%, which was significantly higher than seeds inoculated with *F. thapsinum* 77%*, A. alternata* 76%, *E. sorghinum* 78%, *C. lunata* 76% and uninoculated seeds 92% (Table 3). Seedling emergence (%) under greenhouse conditions was significantly influenced by target pathogens. The seed inoculated with *F. thapsinum* reduced the seedling emergence to 68% followed by *A. alternata* 70%, *C. lunata* 72%, and *E. sorghinum* 75%. However, a significantly higher seedling emergence of 98% was recorded in seeds treated with *E. alba* extract (Supplementary Figure S1), compared to control (Table 3). The percentage of infected seedlings at 10 and 30 days after inoculation was significantly higher in *F. thapsinum* (62%) followed by *C. lunata* (61%), *A. alternata* (60%), and *E. sorghinum* (58%). Whereas when seeds received *E*. *alba* treatment, it significantly reduced the infection by 90% compared to untreated infected seeds (Table 3). Significantly higher shoot length (32 cm), root length (10 cm), shoot dry weight (2 g), and root dry weight (0.17 g) were noticed in the *E. alba* treated seeds over untreated control.

The field trial results showed a significant improvement in the seedling emergence of 98% in the seeds treated with *E. alba* extract compared to that of negative control (84%). Significantly higher disease protection of 66% was observed in the *E. alba* treated seed compared to control (44%). 1000 seed weight was significantly higher in the *E. alba* treatment (20.8 g) compared to that of negative control (Table 4 and Supplementary Figure S2a,b).

**Figure 3:**
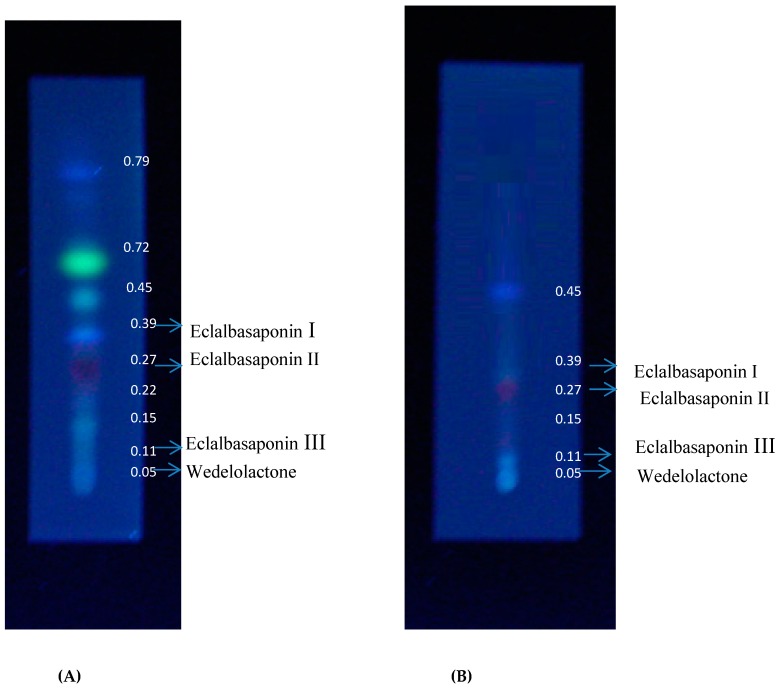
Thin layer chromatography (TLC) profile of *E. alba* (**A**) methanolic crude and (**B**) column purified extract.

**Figure 4:**
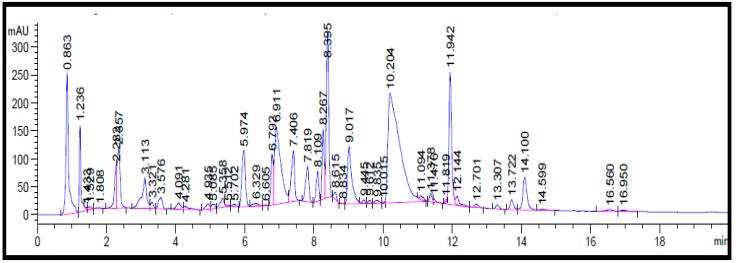
Ultra-performance liquid chromatography (UPLC) Chromatogram profile of *E. alba* crude extract at 260 nm.

**Figure 5:**
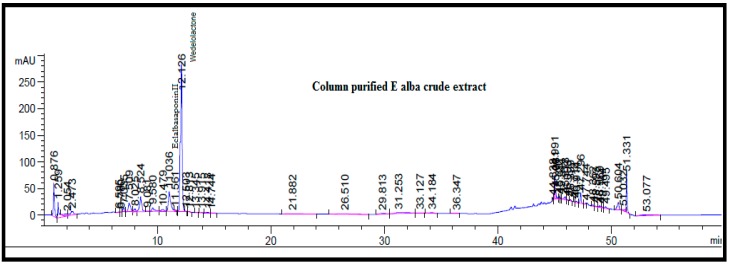
UPLC chromatogram profile of the column purified fraction of *E. alba* at 260 nm.

Phytochemicals in the methanol fraction of *E. alba* were studied using various chromatographic techniques such as TLC, UPLC-DAD, UPLC-MS/MS, and ^1^H-NMR. The TLC revealed the presence of nine major spots with distinct Rf values (Figure 3). The chromatographic profile of crude extract and purified methanol fraction were obtained by UPLC reverse phase method. Nine major peaks were identified in the purified methanol fraction compared to 32 unidentified peaks in crude. The presence of three major categories of compounds, such as saponins, coumestans, and flavonoids, in column purified sample was confirmed based on the Relative Retention Time (RRT) with respect to wedelolactone (Table S1, Figure 4 and Figure 5).

The more precise chemical identification and characterization of the major bioactive compounds were done by using UPLC triple quadrupole mass spectrometry with electrospray ionization (ESI) interface. Ten major compounds were identified, out of which four compounds could be identified as saponins, coumestans, and flavonoids based on MS features (Supplementary Figure S3). The identified compounds with their concentration (area abundance %), and retention time (RT) are represented in Table 5 and Supplementary Figure S4. 

**Figure 6:**
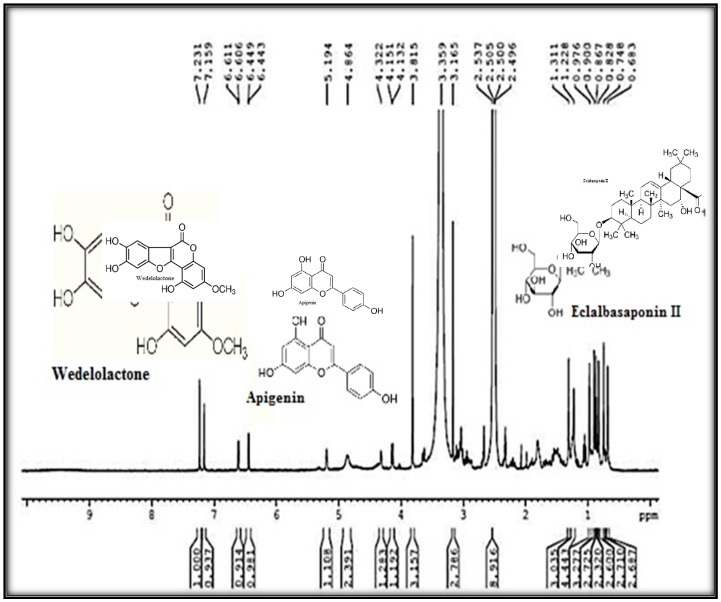
Proton NMR spectra of column-purified antifungal fraction of *E. alba.*

Based on the mass to charge ratio (*m*/*z*), the compounds showed [M-H]^−^ at *m*/*z* 349 and *m*/*z* 269 which corresponds to apigenin sulfate and apigenin. Since the molecular masses of triterpenoid saponins are usually large, we used two collision energies (CE) for identification. The signals recorded at *m*/*z* 797.9, *m*/*z* 841.3, and *m*/*z* 875.3 were identified as [M-H]^−^ corresponding to eclalbasaponin II, ecliptasaponin C, and eclalbasaponin VI, respectively. The wedelolactone was identified by comparing its retention time and *m*/*z* value (313.1) with respect to the reference standard (Supplementary Figure S4). The compounds identified in MS were further confirmed by structural elucidation using 400 MHz ^1^H-NMR (Figure 6). The singlets at δ 7.23, 7.16 ppm and doublets at δ 6.61, 6.61 ppm and 6.45 and 6.44 ppm suggested the presence of phenolic compounds, such as wedelolactone, and flavonoids, such as apigenin. The spectrum presented one proton broad singlet at δ 5.19 ppm, which indicated the presence of olefins proton. Intensities of three protons with δ 0.68, 0.75, 0.83, 0.86, 0.90, 0.97 singlets and 1.31 ppm indicated the presence of tertiary methyl groups and a proton signal at δ 4.32 revealed the presence of the glucose moiety of eclalbasaponins.

## 4. Discussion

Sorghum is a staple food for the majority of the economically poor population in India and African countries. The pathogenic fungi lead to loss of germination and low grain yield, thus, posing a severe threat to the economic resources of poor farmers. Further, the fungal infestation in seeds manifests mycotoxin posing health risks and reduces the nutritional quality of grains and makes the grains unfit for human consumption. Several chemical fungicides are available, but the unscientific and indiscriminate use of these fungicides over the years has led to resistance in pathogens and poses environmental risks. Further, many farmers cannot afford to use chemical fungicides because of their high cost. This necessitates the investigation of alternative disease control measures, such as botanical fungicides. Seed treatment with *E. alba* extract has already been shown to reduce fungal infection, enhance growth, and increase the yield of sorghum [20,21,22,23]. In this context, the in vitro antifungal potential of pharmacologically important *E. alba* on economically destructive sorghum grain mold pathogens was evaluated.

Freshly collected *E. alba* was powdered and extracted sequentially with different polarity solvents by the soxhlet extraction process. Among all the solvent extracts, methanol extract exhibited the highest antifungal activity on target pathogens *E. sorghinum, F. thapsinum, C. lunata,* and *A. alternata*. The antibacterial, antifungal, and antiviral activity and pharmacological use of *E*. *alba* extract were reported by several researchers. The herbal extract zone of inhibition was shown to be equal to or larger than the zone of inhibition of synthetic chemicals [24]. 

The crude *E. alba* extract was further purified using column chromatography to remove the possible inactive phyto-compounds and pigments, which could interfere with the antifungal activity. The extract was fractioned based on differential polarity by silica column and the tested for antifungal activity. The methanol fraction exhibited better antifungal potential compared to the crude extract. The elimination of the hexane and ethyl acetate soluble fraction improved the potency of the methanol fraction compared to the crude extract.

The treatment with *E. alba* extract significantly influenced the germination of sorghum seed. The in vitro germination was evaluated by the between paper method, and a significant difference in germination percentage was observed among the treatments. The germination percentage of *E. alba* treated seeds remained statistically on par with Bavistin treated seeds. The present study has clearly demonstrated that the seeds treated *E. alba* extract have improved germination and seedling emergence by suppressing the pathogens. The lower infection could be due to the presence of antifungal metabolites in *E. alba* that suppress the growth of pathogenic seed-borne fungi. Increased disease protection of *E. alba* was observed under greenhouse studies, whereas reduced efficacy was noticed in field conditions, which may be due to the leaching the *E. alba* extract from treated seeds in soil. Further, the stability and persistence of the extract may be a point to address to increase the disease protection in field conditions. With this background, the decision has been taken to formulate the *E. alba* extract with a biodegradable polymer and penetrating agent. The improved disease protection in field conditions was obtained by using the *E. alba* formulations. *Lawsonia inermis* and *Psoralea corylifolia* botanical formulation were prepared based on antifungal tests and greenhouse studies against purple blotch in onion and damping off and Alternaria blight in tomato. The formulation resulted in an increased yield of tomato and onion with reduced disease incidence up to 63% and 64% [25]. Formulation of *Datura metel* extract along with *Pseudomonas fluorescens* and *Bacillus subtilis* reduced Fusarium wilt disease incidence caused by *Fusarium* *oxysporum* under greenhouse and field conditions with an increased yield of banana [26]. Additionally, *E. alba* extract proved to be non-toxic towards Sprague Dawley rats and New Zealand white rabbits in oral administration, acute dermal irritation, and eye irritation studies in vitro [4]. Formulations from the study induced significant protection against grain mold diseases. The broad spectrum antifungal activity and efficacy of the purified methanol fraction was investigated for its chemical composition using various chromatographic techniques. The TLC profiling of the purified fraction revealed the presence of saponins, coumestans, and flavonoids which were confirmed by comparing the Rf value with the literature reported values. The results are in line with the phytochemical screening of *E. alba* methanolic extract and revealed the presence of flavonoids, tannins, saponins, coumestans, and alkaloids [27]. The UPLC chromatographic profile also supported the findings of TLC. The presence of three major categories of compounds, such as saponins, coumestans and flavonoids, in the column purified sample were identified based on the Relative Retention Time (RRT) with respect to wedelolactone. The *E. alba* phytochemical constituents contained triterpenoid saponins, flavonoids, thiophenes, and steroids [8]. 

Aqueous extract and methanol extract of *E. alba* inhibited the mycelial growth of *Drechslera* *halodes*, *Aspergillus niger*, *F. oxysporum*, *Aspergillus flavus*, *Fusarium solani,* and *Fusarium moniliformae* by poisoned food technique [28]. Seed treatment with essential oil of *Cymbopogon giganteus*, *C nardus* and *C schoenanthus* showed reduced incidence of *Curvularia* sp., *Fusarium* sp., *Phoma* sp., *Colletotrichum* sp. in sorghum and pearl millet [29]. The saponin fraction obtained from *E. alba* exhibited antimycotic activity against *A. niger, A. flavus* and *A. fumigatus* pathogens and the inhibition was on par with antibiotics, such as Amphotericin-B and chloramphenicol [30]. The presence of coumestans and wedelolactone in the ethyl acetate fraction of *E. alba* showed antimicrobial property against *Salmonella typhimurium, Staphylococcus epidermidis*, *S. aureus*, *Shigella flexneri* and antifungal activity against *Aspergillus ochraceus* [5,31]. Flavonoid content of *E. alba* inhibited human pathogens, such as *Proteus mirabilis, Bacillus subtilis, Pseudomonas fluorescens,* and *Staphylococcus aureus* [32]. Wedelolactone from *E. alba* play an important role in the inhibition of *S. typhimurium* and *S. epidermidis,* and this compound can be used in treating the above pathogen infections in humans [33]. Eclalbasaponin from *E. alba* exhibited antibacterial activity against *P. aeruginosa* and *B. subtilis* by disrupting the cell wall of bacteria resulting in loss of bacterial cell viability [24]. 

The precise identification of the potential antifungal bioactive metabolites in the purified fraction was investigated by UPLC coupled mass spectrometry finger printing. The total ion chromatograph of the extract and mass to charge ratio (*m*/*z*) revealed the presence of apigenin sulfate, apigenin, eclalbasaponin II, ecliptasaponin C, eclalbasaponin VI, and wedelolactone. The presence of eclalbasaponin II and wedelolactone was confirmed by the chemical shift value of NMR.

The chemical characterization of bioactive metabolites revealed the presence of saponins with higher concentrations of eclalbasaponin II and wedelolactone in the purified methanolic fractions of *E. alba* may lead to loss of pathogen cell viability. Saponins are secondary metabolites stored as inactive precursors in plant cells, when pathogen infects the plant it will alter into a biologically active antibiotic through enzymes [34,35]. Saponins along with sterols form a complex in the membrane of fungi and cause a break in the integrity of the cell membrane by inducing transmembrane pores [36,37]. α-Tomatine, a saponin isolated from *Lycopersicon esculentum* can induce cell death in *F. oxysporum* by activation of G-protein signaling pathway and tyrosine kinase leading to elevation in intracellular Ca^2+^ and reactive oxygen species accumulation [38]. Saponins possess antifungal, antiulcer, antiviral, antibiotic, anti-inflammatory, and hepatoprotective activities [39]. Triterpenic saponin isolated from *Diploknema butyracea* and *Sapindus mukorossi* showed antifungal activity against *Rhizoctonia bataticola* and Sclerotium rolfsii fungal pathogens [40]. The saponin rich extract obtained from *Quillja saponaria* bark, *Yucca schidigera,* and *Balanites aegyptiaca* fruit mesocarp, showed better antifungal activity against *Pythium ultimum*, *Colletotrichum coccodes*, *Alternaria solani,* and *Verticillium dahlia* [41]. The saponin isolated from *E. alba* causes disruption of microbial cell membrane resulting in loss of microbial cell viability [42]. Morphological deformation of the *Pyricularia oryzae* mycelia was observed due to eclalbasaponin II isolated from *E. alba* [43]. 

## 5. Conclusions

The results demonstrated antifungal activity of the methanol fractions of *E. alba* on sorghum pathogens, such as *F. thapsinum*, *A. alternata, E. sorghinum* and *C. lunata*. The elimination of the nonpolar soluble from the methanol extract by column purification, improved the antifungal potency of the methanol *E. alba* extract. The chemical characterization of the bioactive antifungal metabolites revealed the presence of phyto-analogs, such as eclalbasaponin II and wedelolactone, at higher concentrations in methanolic factions of *E. alba*, which significantly contributed to the antifungal activity against target sorghum pathogens. Further, this work substantiated the findings of earlier work [21,22] which has shown the antifungal activity of *E. alba* extracts against sorghum pathogens. The present study shed light on the use of methanolic *E. alba* fractions as a potential alternative and cost effective botanical fungicide on economically destructive sorghum grain mold pathogens. 

## Figures and Tables

**Table 1:** Antifungal activity of methanolic extract of *Eclipta alba* (Agar well diffusion assay).

	Zone of Inhibition (mm)			
Target Pathogens	*F. thapsinum* ^$^	*E. sorghinum* ^$^	*A. alternata* ^$^	*C. lunata* ^$^
Methanolic crude extract (120 mg/mL)	5.8	6	5.9	5.9
Purified extract (120 mg/mL)	6.2	6.8	6.6	6.5
Bavistin (1 mg/mL)	7.3	6.9	7	7.1
Mean	6.4	6.6	6.5	6.5
SD	±2.8	±2.9	±2.8	±2.7
* CD (0.05 ^#^)	0.29	0.22	0.24	0.56

* CD—critical difference, SD—Standard deviation, ^#^—5% level of Significance, ^$^—Three replications.

**Table 2:** Percentage growth inhibition exhibited by *E. alba* extract by Microtiter plate assay.

	Target Pathogens (Percent Growth Inhibition)			
Concentration of *E. alba* Extract (µg/mL)	*F. thapsinum*	*E. sorghinum*	*A. alternata*	*C. lunata*
20	34	34	33	30
40	42	46	41	48
60	52	64	56	63
80	73	74	69	74
100	80	82	86	83
120	86	87	89	88
Bavistin (1 µg/mL)	91	91	94	93
Mean	65	68	67	68
SD	±1.91	±6	±3	±3
* CD (0.05 ^#^)	2.11	2.32	2.38	2.05

* CD—critical difference, SD—standard deviation, ^#^—5% level of Significance.

**Table 3:** Influence of purified *E. alba* methanol extract on sorghum pathogens and growth parameters under greenhouse condition.

Treatment	In vitro Germination. (%)	Seedling Emergence Rate (%)	Disease Incidence at 10th Day	Disease Incidence at 30th Day	Shoot Length (cm)	Root Length (cm)	Dry Shoot Weight (g)	Dry Root Weight (g)
Control	*92*	84	34	36	23.30	6.80	1.15	0.10
*F. thapsinum*	*77*	68	62	66	11.87	5.60	0.82	0.10
*E. sorghinum*	*78*	75	58	61	11.93	4.60	0.84	0.10
*A. alternata*	*76*	70	60	62	11.97	4.33	0.75	0.10
*C. lunata*	*76*	72	61	66	11.57	4.17	0.67	0.10
*E. alba* extract + *F. thapsinum*	*99*	97	5	11	30.67	10.50	2.10	0.18
*E. alba* extract +*E. sorghinum*	*99*	98	9	9	31.23	10.67	2.12	0.18
*E. alba* extract +*A. alternata*	*97*	99	15	15	33.97	9.37	2.04	0.16
*E. alba* extract + *C. lunata*	*98*	98	5	5	33.93	9.03	2.13	0.17
Bavistin + *F. thapsinum*	*98*	98	5	5	31.97	11.07	2.08	0.18
Bavistin + *E*. *sorghinum*	*97*	99	9	9	30.37	10.17	2.16	0.18
Bavistin + *A. alternata*	*98*	99	1	1	32.90	10.80	2.01	0.15
Bavistin + *C. lunata*	*99*	97	1	1	33.67	9.43	2.07	0.15
*E. alba* extract	*98*	98	4	5	31.45	10.9	2.12	0.17
Bavistin	*99*	99	1	1	33.98	11.12	2.18	0.18
Mean	92.07	90.07	22.00	23.53	26.32	8.57	1.68	0.15
SD	±9.72	±12.38	±25.22	±26.53	±9.41	±2.68	±0.62	±0.04
* CD (0.05 ^#^)	4.37	5.36	8.11	8.03	1.68	1.31	0.18	0.03

* CD—critical difference. %—percentage. cm—centimeter. g—gram. ^#^—5% level of Significance.

**Table 4:** Influence of purified *E. alba* methanol extract on sorghum pathogens and growth parameters under field conditions.

Treatment	Disease Protection at 60 DAS (%)	% of Emerged Seedlings at 10 DAS	Plant Length	Panicle Length (cm)	Panicle Width (cm)	1000 Seed
Control	44	84	105	16.2	6	16.85
*F. thapsinum*	19	68	101	13.1	5.1	14.37
*E. sorghinum*	20	75	100	13	4.2	13.23
*A. alternata*	20	70	101	12.2	5.2	13.0
*C. lunata*	21	72	100	13.1	4.0	11.23
*E. alba* extract + Pathogen suspension	66	99	110	17.0	6.4	20.8
*E. alba* extract	80	98	110	17.0	6.6	21.0
Bavistin	89	98	112	19.0	6.7	21.2
Mean	44.9	83.0	104.9	15.1	5.53	16.5
SD	±29.53	±13.55	±5.08	±2.52	±1.06	±4.07
CD (*p* < 0.05 ^#^)	5.61	5.8	3.98	1.21	0.27	0.97

CD—critical difference, DAS—Days after sowing, ^#^—5% level of Significance.

**Table 5:** Percent abundance of the compounds in the column purified methanol fraction of *E. alba* analysed using ultra-performance liquid chromatography-mass spectroscopy (UPLC-MS/MS).

Sl. No	RT	*m*/*z*	Probable Compounds	% Abundance
1	3.17	349.0	Apigenin sulfate	1.25
2	8.91	447.0	Luteolin-7-O-*β*-D glucoside	0.37
3	11.58	285.9	Luteolin	0.85
4	11.58	364.9	Luteolin sulfate	0.45
5	12.08	797.9	Eclalbasaponin II	17.20
6	12.21	313.1	Wedelolactone	11.80
7	13.56	269.0	Apigenin	6.41
8	17.61	957.3	Eclalbasaponin III	0.17
9	19.67	841.3	Ecliptasaponin C	2.01
10	20.86	875.3	Eclalbasaponin VI	10.20

## Supplementary Materials

The following are available online at https://www.mdpi.com/2223-7747/8/3/72/s1, Figure S1: Effect of seed treatment with *E. alba* extract (120mg/mL) on emergence of seedlings for seeds infected with (A) Pathogen control (*F. thapsinum*) (B) Water treated control (C) Bavistin treated (D) *F. thapsinum* (E) *E. sorghinum* (F) *A. alternata* (G) *C. lunata*, Figure S2: (A) Effect of seed treatment on performance of sorghum in field trials by randomized block design (30 DAS), (B) Effect of seed treatment on performance of sorghum in field trials by randomized block design (60 DAS), Figure S3: UPLC MS/MS full scan m/z 100 -1700 spectra of *E. alba* column purified sample, Figure S4: Mass chromatography data of (A) Apigenin, (B) Wedelolactone, (C) Eclalbasaponin II, and (D) EclalbasaponinVI, Table S1: Major peaks retention time and peak area of column purified fraction of *E. alba* at 260 nm.
