# Supplementary material for: Antifungal Activity of Eclipta alba Metabolites against Sorghum Pathogens
Source: Plants (Basel). 2019 Mar 22;8(3):72. doi: 10.3390/plants8030072 (PMC6473702; doi:10.3390/plants8030072)
Supplement: Supplementary file 1 [file plants-08-00072-s001.pdf]

Supplementary

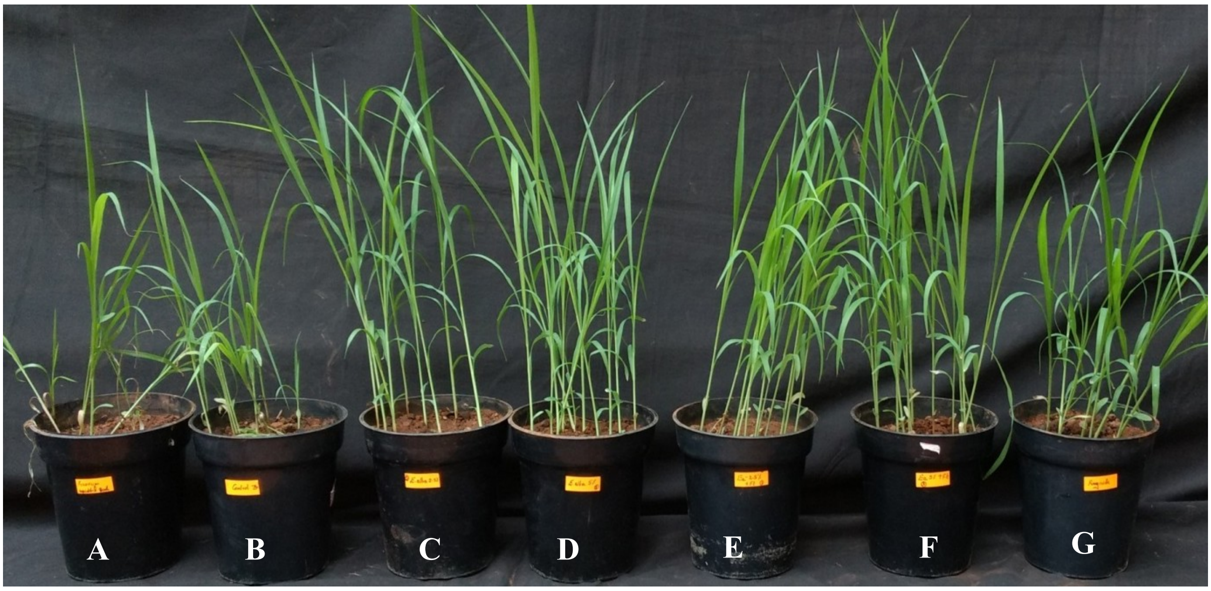

Figure S1. Effect of seed treatment with *E. alba* extract (120mg/ml) on emergence of seedlings for seeds infected with **A**. Pathogen control (*F. thapsinum*) **B**. Water treated control **C**. Bavistin treated **D**. *F. thapsinum* **E**. *E. sorghinum* **F**. *A. alternata* **G**. *C. lunata.*

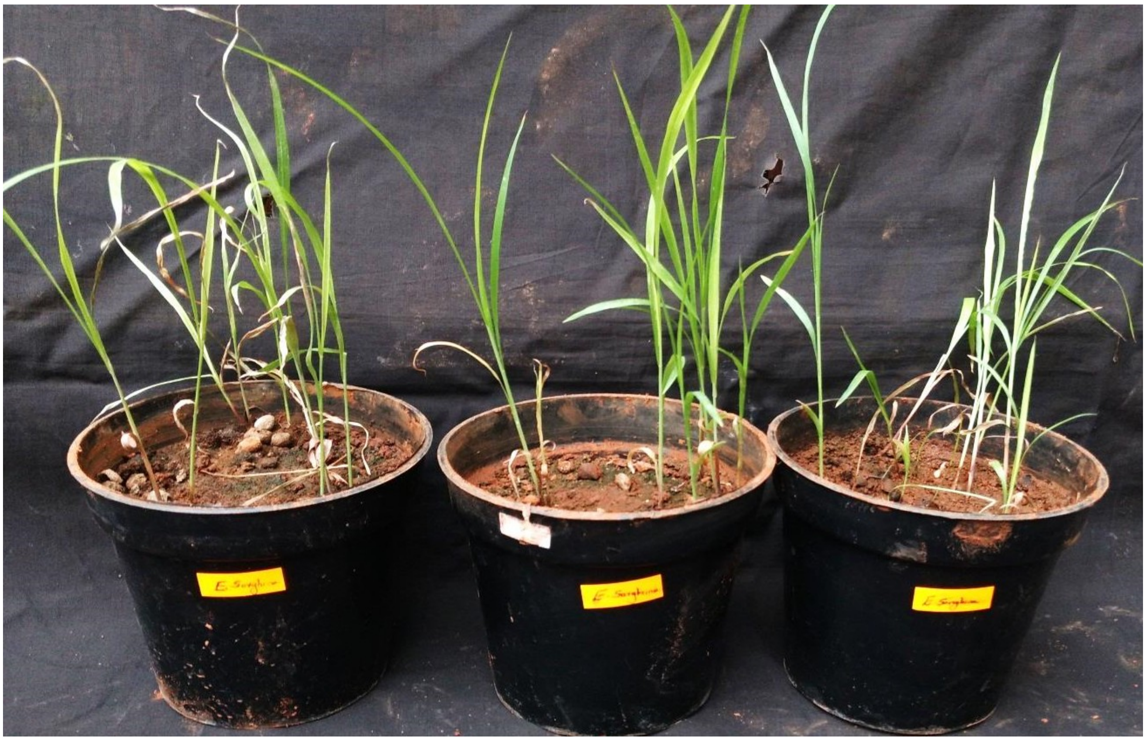

Figure S1a. *F.thapsinum* infected plants.

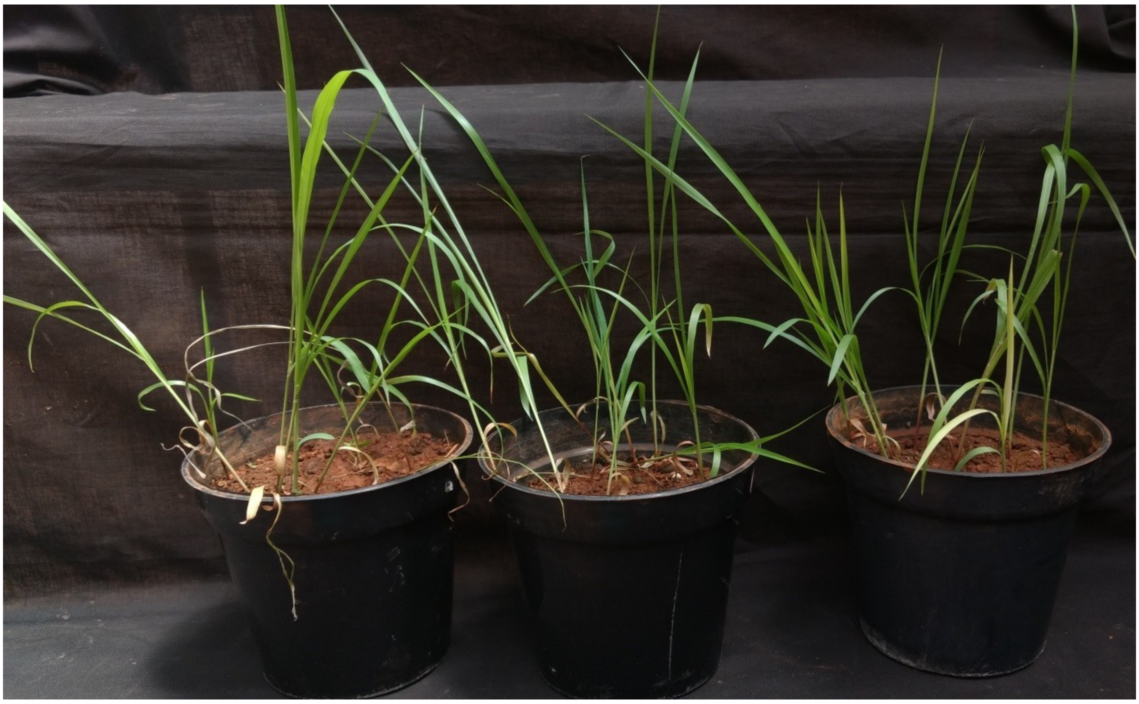

Figure S1b. *E. sorghinum* infected plants.

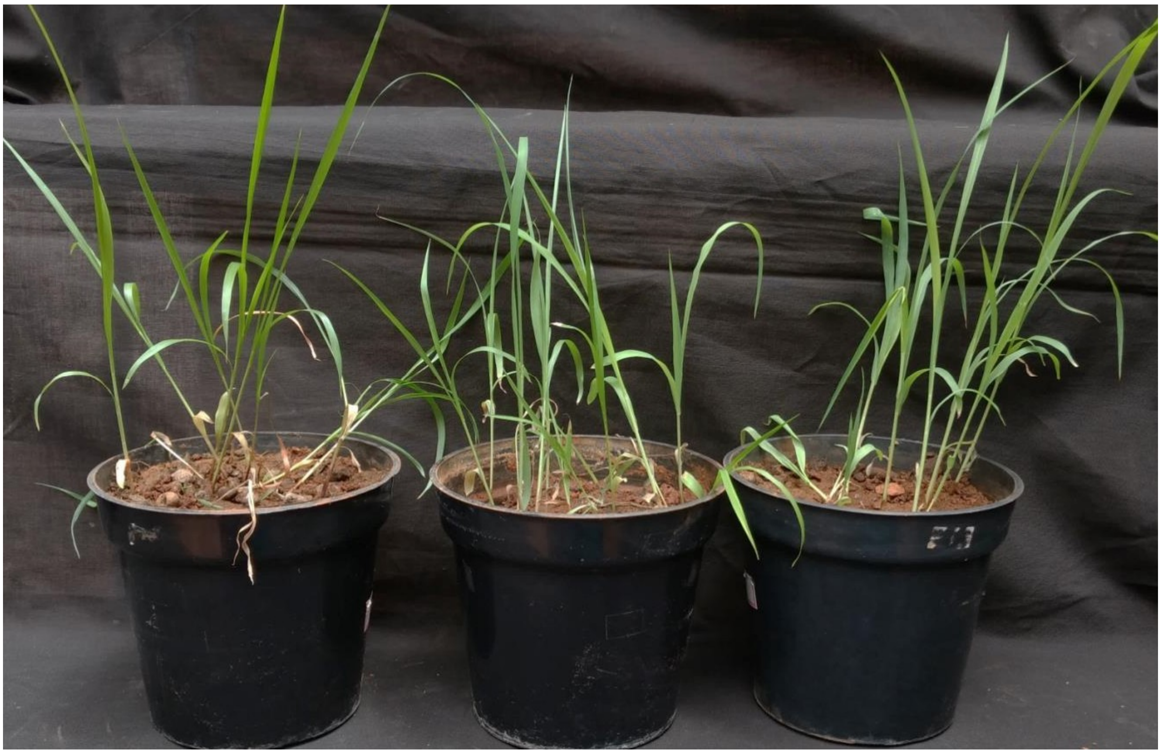

Figure S1c. *A. alternata* infected plants.

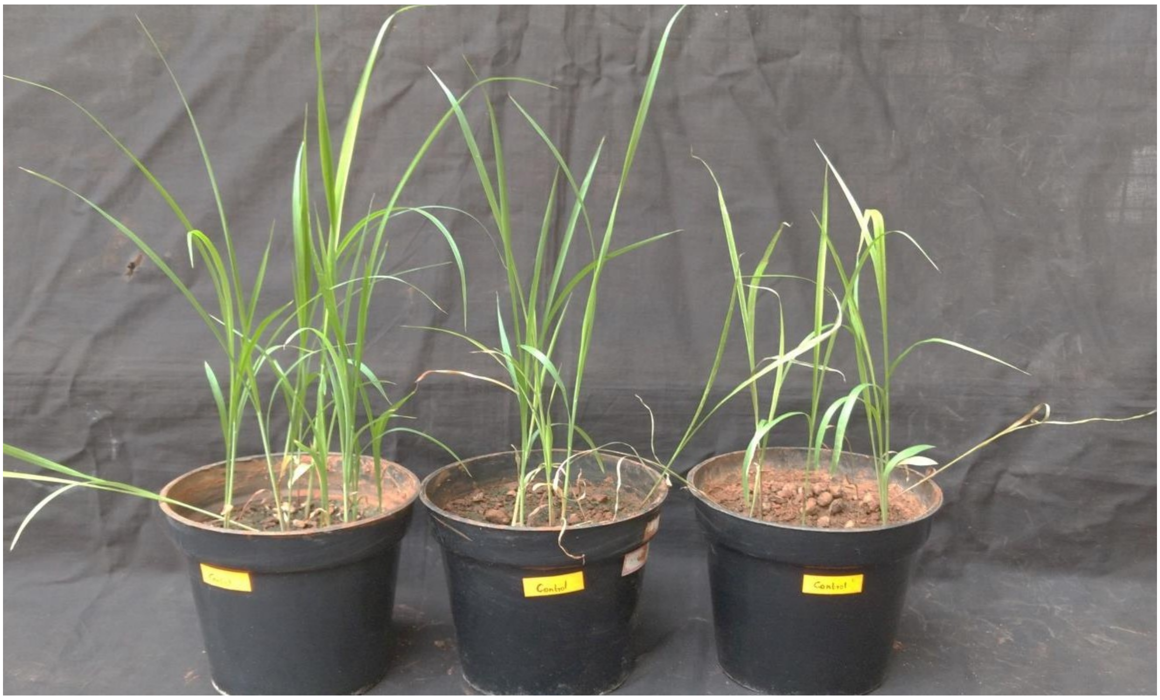

Figure S1d. *C. lunata* infected plants.

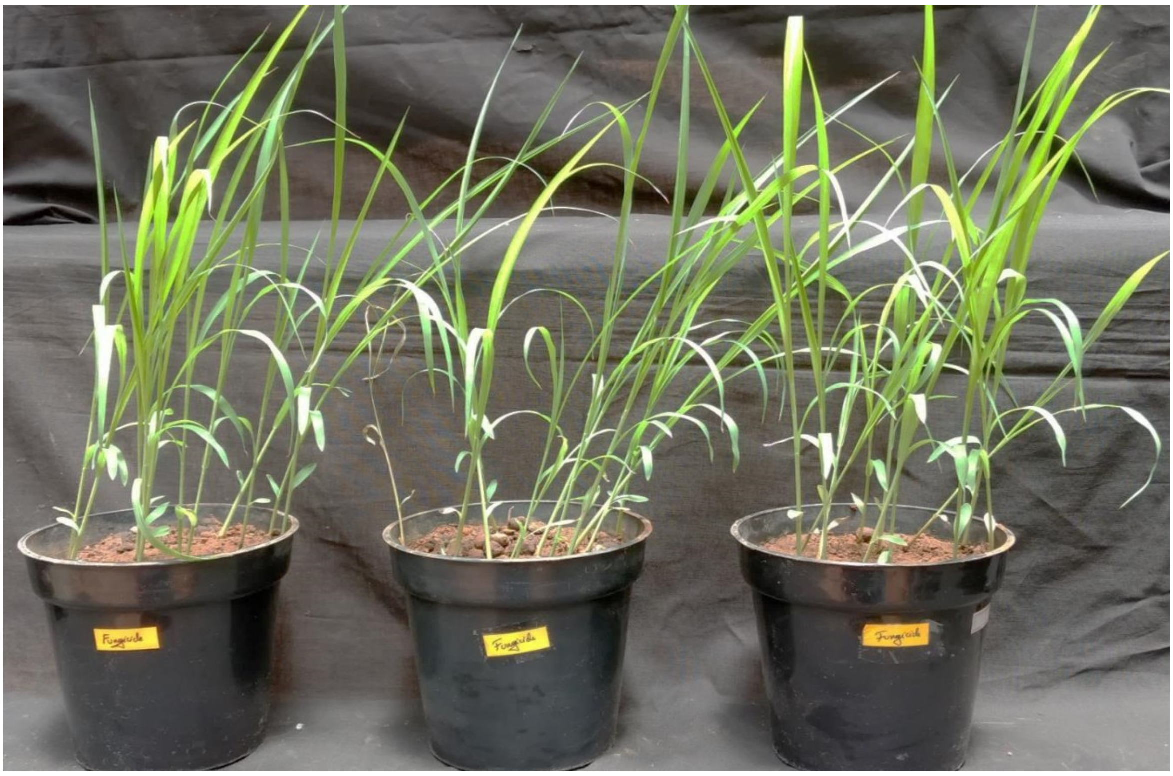

Figure S1e. Fungicide treated plants.

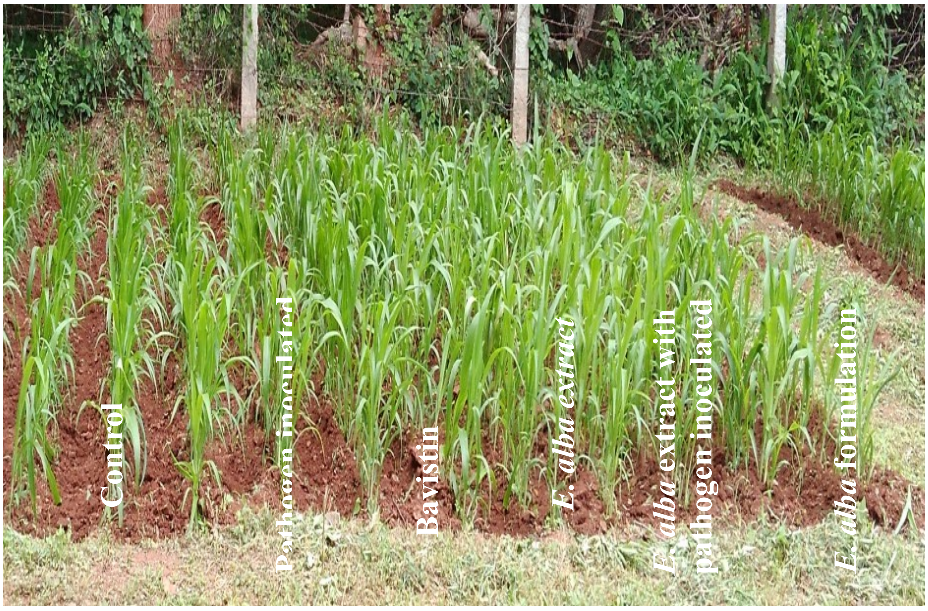

Figure S2. (a). Effect of seed treatment on performance of sorghum in field trials by randomized block design (30 DAS).

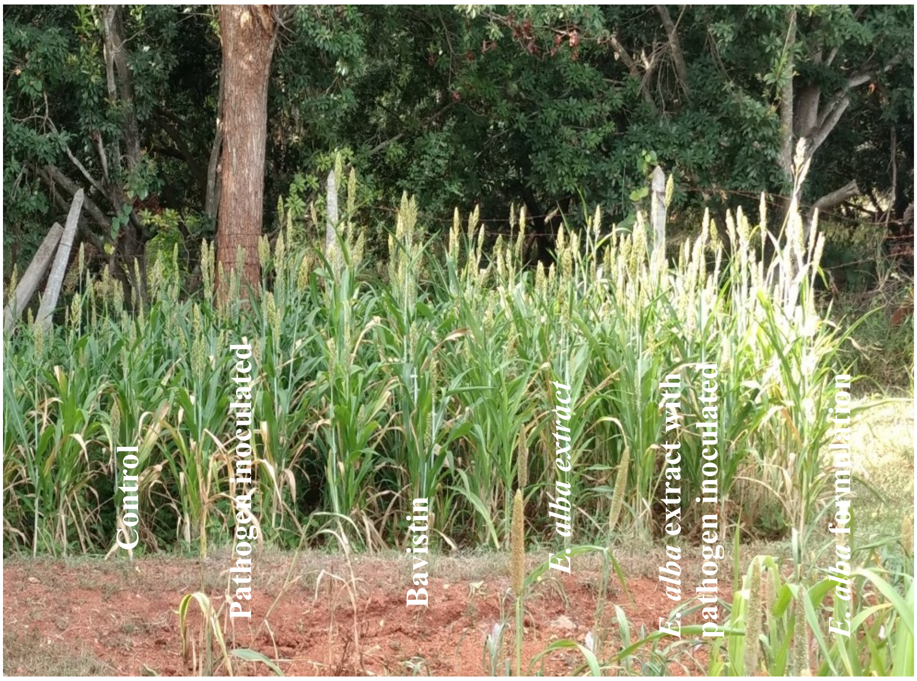

Figure S2. (b). Effect of seed treatment on performance of sorghum in field trials by randomized block design (60 DAS).

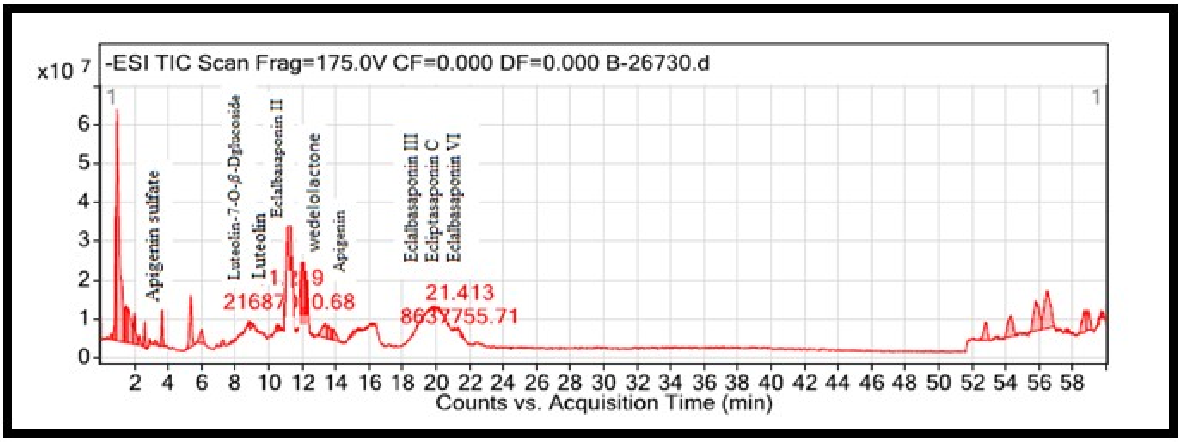

Figure S3. UPLC MS/MS full scan m/z 100–1700 spectra of *E. alba* column purified sample.

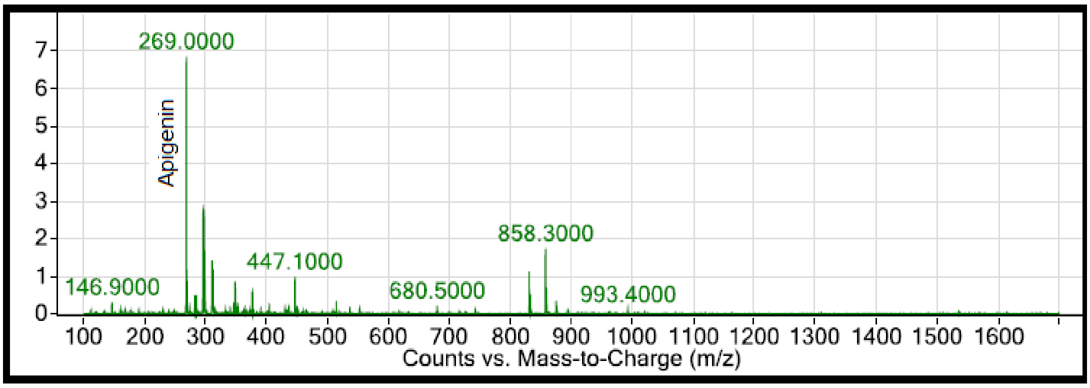

(a)

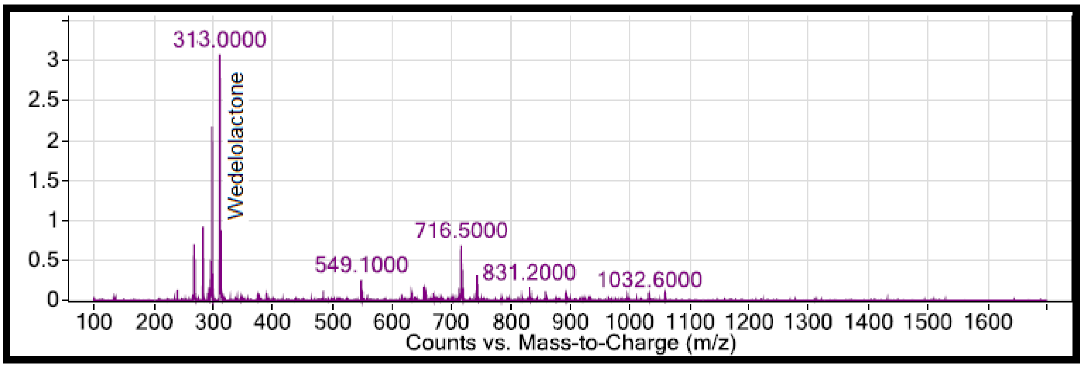

(b)

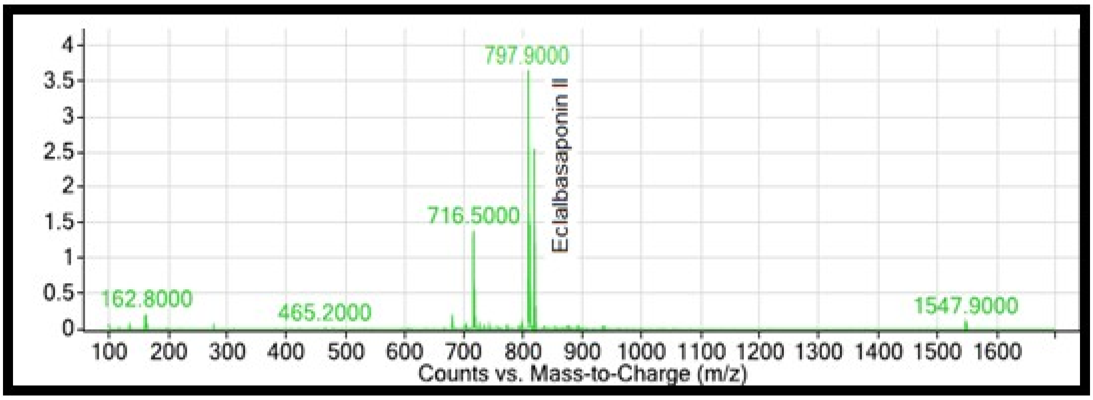

(c)

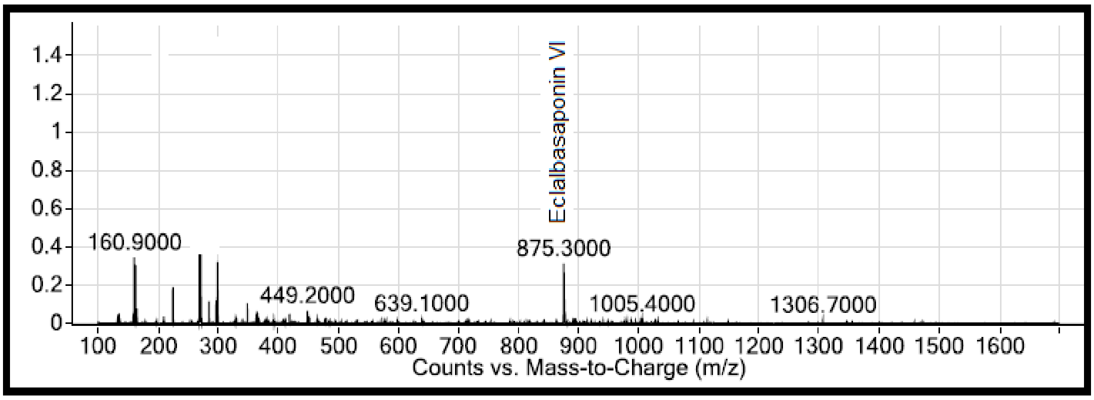

(d)

Figure S4. Mass chromatography data of (**a**) Apigenin, (**b**) Wedelolactone, (**c**) Eclalbasaponin II and (**d**) EclalbasaponinVI.

Table S1. Major peaks retention time and peak area of column purified fraction of. *E. alba* at 260 nm.

Sl No	Retention # Time	Peak Area	RRT *
1	0.88	394.80	0.08
2	1.26	279.44	0.11
3	2.05	79.65	0.18
4	2.47	146.84	0.21
5	10.48	54.18	0.91
6	11.04	551.71	0.95
7	11.56	5.82	1.00 (Eclalbasaponin)
8	12.13	2913.02	1.05 (Wedelolactone)
9	12.50	4.60	1.08
10	14.74	4.84	1.28
11	21.88	43.56	1.89
12	26.51	72.57	2.29
13	29.81	53.75	2.58
14	31.25	135.73	2.70
15	33.13	5.84	2.87
16	34.18	27.01	2.96
17	36.35	2.10	3.14
18	50.60	148.59	4.38
19	51.03	47.97	4.41
20	51.33	380.83	4.44
21	53.08	115.23	4.59

RRT *: Relative retention time with respect to Eclalbasaponin, #: The Sample chromatogram subtracted from blank.
